# A pipeline for metabarcoding and diet analysis from fecal samples developed for a small semi-aquatic mammal

**DOI:** 10.1371/journal.pone.0201763

**Published:** 2018-08-14

**Authors:** Oliver Hawlitschek, Angel Fernández-González, Alfonso Balmori-de la Puente, Jose Castresana

**Affiliations:** 1 Institut de Biologia Evolutiva (CSIC-Universitat Pompeu Fabra), Passeig Maritim de la Barceloneta, Barcelona, Spain; 2 Zoologische Staatssammlung München (ZSM-SNSB), München, Germany; 3 Biosfera Consultoría Medioambiental S.L., Calle Candamo, Oviedo, Spain; Oklahoma State University, UNITED STATES

## Abstract

Metabarcoding allows the genetic analysis of pooled samples of various sources. It is becoming popular in the study of animal diet, especially because it allows the analysis of the composition of feces without the need of handling animals. In this work, we studied the diet of the Pyrenean desman (*Galemys pyrenaicus*), a small semi-aquatic mammal endemic to the Iberian Peninsula and the Pyrenees, by sequencing COI minibarcodes from feces using next-generation sequencing techniques. For the identification of assembled sequences, we employed a tree-based identification method that used a reference tree of sequences of freshwater organisms. The comparison of freshly collected fecal samples and older samples showed that fresh samples produced significantly more sequencing reads. They also rendered more operational taxonomical units (OTUs), but not significantly. Our analyses of 41 samples identified 224 OTUs corresponding to species of the reference tree. Ephemeroptera, Diptera excl. Chironomidae, and Chironomidae were the most highly represented groups in terms of reads as well as samples. Other groups of freshwater organisms detected were Plecoptera, Trichoptera, Neuropteroida, Coleoptera, Crustacea, and Annelida. Our results are largely in line with previous morphological and genetic studies on the diet of the Pyrenean desman, but allowed the identification of a higher diversity of OTUs in each sample. Additionally, the bioinformatic pipeline we developed for deep sequencing of fecal samples will enable the quantitative analysis of the diet of this and other species, which can be highly useful to determine their ecological requirements.

## Introduction

DNA barcoding has been established as a simple and reliable way of identifying individual DNA samples of organisms to species level [1–7]. The true power of the application, however, does not lie in the identification of individual samples, which is often also possible using other methods such as morphological or biochemical methods. Instead, its advantage is in the identification of bulk samples that include the DNA of many different species without previously isolating these samples individually, an approach which is termed metabarcoding [8]. Metabarcoding allows the identification of species, e.g., from specimen pools in preservative medium [9], environmental samples (water or soil: [10,11]), or gut and fecal samples [12].

Despite the higher potential of metabarcoding, its adoption is still very limited. As of January 2018, Web of Science lists 5,615 publications on 'barcoding' vs. 592 on 'metabarcoding' or 'meta-barcoding'. One of the reasons for this situation is the relatively recent availability at affordable prices of next-generation sequencing (NGS) technology, which is required to process multiple sequences in parallel. A second reason for the relatively slow adoption of metabarcoding is the requirement of a comprehensive and reliable barcode database. The globally leading barcode database BOLD (Barcode of Life Data Systems, [13]) hosts almost 6 million barcodes of more than 187,000 described species as of January 2018, but this is only a small percentage of the total estimated global biodiversity [14,15]. Furthermore, not all taxonomic groups and geographic regions are equally well represented [16].

Freshwater invertebrates are among the groups that are well represented in BOLD [13,17–19], mostly because of their importance in ecology and conservation biology [20,21]. The amount of data available in Western Europe [22] can be considered suitable for metabarcoding studies, although further improvement of the database in some specific regions is needed. In fact, analyses of environmental samples from European freshwaters have already been published [11]. The availability of this information makes the analysis of fecal samples of species feeding on freshwater invertebrates highly feasible.

Feces are generally a good source of DNA as they are relatively easy to obtain, also from elusive species [23–25]. On the other hand, the quality of the obtained DNA is often much lower than in other samples due to the digestion process before and the decomposition processes after defecation [26]. In studies on the diet of terrestrial herbivores, plant-specific primers can be used to exclude most sources of contamination. In the case of insectivores, many insect species are involved in the decomposition of feces, making the distinction between sequences originating from digested prey and decomposing insects (e.g., eggs of flies) more problematic. Further problems may arise in aquatic or humid environments, where the degree of contamination through environmental DNA (eDNA) or microorganisms may be higher than in dryer environments.

Our study species is the Pyrenean desman (Lipotyphla: Talpidae: *Galemys pyrenaicus*), a small semi-aquatic insectivore mammal endemic to the Iberian Peninsula and the Pyrenees. The Pyrenean desman mostly inhabits lotic waters and is sensitive to pollution and anthropogenic modifications of its habitat structure [27]. Therefore, it is today restricted to relatively well-conserved habitats, mostly in mountainous regions, and is listed as Vulnerable on the IUCN Red List [28]. As a characteristic species of a threatened habitat, the Pyrenean desman is often employed as flagship for conservation measures [29]. Nevertheless, the cryptic habits of this species are the reason why its biology is still not well known [30]. This also includes feeding habits. Previous studies by Santamarina [31], Castién & Gosálbez [32], and Fernández-Salvador et al. [33] employed the morphological examination of gut contents of captured specimens. These pioneer works already provided highly valuable information on the diet of *G*. *pyrenaicus*. However, morphological studies of gut contents present several problems. Apart from the obvious disadvantage of these invasive techniques, the identification of prey items is often possible only to family or genus level because important characters for morphological identification are lost in digestion [26]. Metabarcoding of fecal samples presents a potential solution to these problems because sampling is non-invasive, requires significantly less effort, and prey items may be reliably identified to species level provided a comprehensive reference library is available [34,35]. Recent genetic studies on the diet of the Pyrenean desman [36–38] already employed a metabarcoding approach. These works were based on BLAST database searches alone, which allow an accurate identification at the species level but have more difficulties with the identification at higher taxonomic ranks due to inherent limitations of this method derived from heterogeneity of evolutionary rates [39–41].

Here we studied the utility of metabarcoding based on the sequencing and analysis of a large number of reads per sample to provide a reliable quantification of the diet of *G*. *pyrenaicus*. To increase the proportion of identified reads, we used a tree-based identification method of the assembled sequences in addition to the BLAST method. We also used fresh and old excrements of *G*. *pyrenaicus* in order to understand if degradation of the excrements may affect the assessment of the diet. We finally provide a full laboratory protocol and a computational pipeline for the analysis.

## Material and methods

### Sampling

Fecal samples of *Galemys pyrenaicus* were obtained from several rivers in Spain in the months of May to November from 2010 to 2015 (Table 1). Most samples used here were collected from two rivers in Zamora, Tera and Tuela. In these two rivers we had the opportunity to use 22 samples that were fortuitously obtained after defecation by specimens that were captured as part of an independent survey work on the species in the Zamora region. To minimize stress, captured specimens were housed in buckets for the period of time strictly necessary to take data and implant a transponder (normally a few minutes). During this time most specimens spontaneously defecated and their release in the river was never delayed to obtain an excrement sample. We refer to samples obtained this way as 'fresh' samples. Additionally, 25 'old' samples were obtained by searching in river banks and stones in the middle of rivers for desman latrines [42,43]. All samples were directly preserved and stored in absolute ethanol. Therefore, fresh samples had a maximum 'age' of a few minutes between defecation and preservation, whereas old samples had unknown 'ages' (several days or, more rarely, weeks) during which they may have been subject to decomposition, erosion, and contamination by organic material from the environment.

**Table 1:** A list of attributes of all samples based on Illumina MiSeq sequencing and metabarcoding analysis. Only the 41 samples for which library amplification was successful are listed. System = Major river system, given for the Zamora samples; Reads = total reads assigned to the sample; % target = reads forming clusters of more than 10 reads that were successfully identified in the tree-based approach; H' = Shannon diversity index.

Sample	Fresh?	Date	River/Locality	System	Region	Reads	% target	OTUs	H'
**BC0023**	yes	27/05/2014	Mondera	Tera	Zamora	187750	85.89	36	0.18
**BC0024**	yes	19/05/2015	Tuiza	Tuela	Zamora	167956	59.82	8	0.08
**BC0028**	yes	02/09/2014	Tuiza	Tuela	Zamora	64908	60.71	16	1.22
**BC0041**	yes	06/11/2013	Curricha	Tuela	Zamora	157264	0.30	5	1.18
**BC0061**	yes	29/05/2014	Parada	Tera	Zamora	137299	67.90	17	0.27
**BC0240**	yes	01/09/2014	Arrochas	Tuela	Zamora	123821	8.89	14	1.46
**BC0293**	yes	01/09/2014	Arrochas	Tuela	Zamora	141171	84.84	36	0.87
**BC0648**	no	21/07/2014	Yuso	-	León	47626	1.90	6	0.79
**BC0780**	no	21/07/2014	Lechada	-	León	71798	32.64	10	0.61
**BC0834**	no	01/09/2014	Arrochas	Tuela	Zamora	104049	73.96	15	1.36
**BC0849**	no	01/09/2014	Arrochas	Tuela	Zamora	26473	26.51	20	1.46
**BC0857**	no	11/09/2014	Castro	Tera	Zamora	35716	3.48	8	1.60
**BC0861**	no	02/09/2014	Tuiza	Tuela	Zamora	151022	7.87	9	0.86
**BC0868**	yes	20/05/2015	Arrochas	Tuela	Zamora	165393	86.91	24	0.90
**BC0873**	no	03/09/2014	Tuela	Tuela	Zamora	135386	20.10	8	0.37
**BC0877**	no	03/09/2014	Tuela	Tuela	Zamora	82208	46.11	14	1.07
**BC0899**	no	11/09/2014	Castro	Tera	Zamora	111970	59.08	17	0.19
**BC0917**	no	03/09/2014	Tuela	Tuela	Zamora	144577	76.54	26	0.70
**BC0939**	no	01/09/2014	Arrochas	Tuela	Zamora	122615	37.39	21	0.22
**BC0943**	no	02/09/2014	Tuiza	Tuela	Zamora	154772	1.23	5	0.34
**BC0974**	no	01/09/2014	Arrochas	Tuela	Zamora	77147	51.40	29	0.36
**BC0981**	no	10/09/2014	Los Tornos	Tera	Zamora	100335	18.25	19	1.14
**BC1035**	yes	28/05/2014	Cabril	Tera	Zamora	17301	1.88	7	1.76
**BC1041**	yes	10/06/2015	Tornos	Tera	Zamora	126101	77.50	20	1.31
**BC1059**	yes	12/06/2015	Parada	Tera	Zamora	184014	80.66	17	0.06
**BC1062**	yes	14/09/2014	Porto	Tuela	Zamora	124906	0.24	2	0.55
**BC1101**	yes	04/06/2015	Cabril	Tera	Zamora	213479	86.15	17	0.22
**BC1108**	yes	09/06/2015	Cabril	Tera	Zamora	138976	73.91	21	0.62
**BC1123**	yes	15/09/2014	Mondera	Tera	Zamora	219398	88.38	16	0.02
**BC1144**	yes	15/09/2014	Mondera	Tera	Zamora	52286	72.83	23	0.54
**BC1150**	yes	16/09/2014	Parada	Tera	Zamora	106664	36.13	17	0.49
**BC1154**	yes	23/09/2014	Parada	Tera	Zamora	117200	0.40	6	1.27
**BC1243**	yes	09/06/2015	Cabril	Tera	Zamora	267589	90.10	29	0.14
**C1131**	no	16/07/2010	Razoncillo	-	Soria	172686	83.91	30	0.81
**C1654**	no	16/07/2010	Razoncillo	-	Soria	118641	18.79	11	1.16
**C1661**	no	16/07/2010	Razon	-	Soria	11146	32.54	7	0.87
**C1671**	no	16/07/2010	Razon	-	Soria	171001	0.12	5	1.28
**C1796**	no	16/07/2010	Razoncillo	-	Soria	118301	0.12	2	0.57
**C3855**	no	06/11/2013	Curricha	Tuela	Zamora	112722	2.54	3	0.20
**C4323**	no	04/10/2014	Romadriu	-	Lleida	9309	1.36	4	1.19
**OHGC001**	no	27/06/2015	Sil	-	Galicia	121419	49.26	14	0.27

### Ethics statement

All captures of Pyrenean desmans and faeces collection were performed with specific permits from the regional government Junta de Castilla y León, which is the environmental competent authority in Spain that can issue permits for these works, where the ethical and scientific-technical requirements are established to perform them with all necessary precautions. Capture permits relevant for this work had registration numbers EENN(ZA)-13/1117-PSR, EENN(ZA)-14/0617-PSR, EENN(ZA)-15/0454-PSR, EENN(ZA)-15/383-JLG and EENN(ZA)-15/366-JLG.

### Molecular laboratory work

We extracted DNA from all fecal samples using the Qiagen DNeasy Blood & Tissue Kit. For maximum yield we extracted the entire volume of every fecal sample (using up to 2 ml of digestion volume). Genetic species identification was conducted for the samples directly collected from the rivers. PCR reactions were set up in a dedicated PCR room that is physically separated from post-PCR working areas and regularly decontaminated by UV-irradiation. For each sample, we amplified either the entire mitochondrial cytochrome *b* gene or one fragment of 278 bp using primers and PCR conditions described in a previous work [43]. PCR products were checked in agarose gels, purified with ExoSAP-IT and sequenced in Macrogen Inc (Seoul, South Korea). It was checked that the sequences obtained corresponded to Pyrenean desman by comparison with previously sequenced haplotypes [43].

The extracted DNA of the samples was then used to prepare a library of the mitochondrial cytochrome *c* oxidase I gene (COI) in three amplification steps: (1) pre-amplification of the standard barcoding fragment of 658 bp (ca. 50 bp from the 5' end of COI) to reduce unspecific amplification, (2) amplicon PCR of a minibarcode of 130 bp, starting 3 bp from the 5' end of the barcoding fragment, to amplify the target fragment and attach part of the Illumina adapters, and (3) index PCR to attach the remaining part of the Illumina adapters and the indices that allowed identification of each sample during subsequent analysis. We used the primers HCO / LCO [44] for the pre-amplification step and Uni-MinibarF1 / Uni-MinibarR1 [45] for the amplicon PCR. We employed a high fidelity polymerase for the index PCR. We ensured that all PCR bands were strong in an agarose gel, with no appreciable differences in intensity between fresh and old samples, and then we pooled all the PCR products. We finally used an agarose gel to purify the target fragment. A detailed library preparation protocol is provided in Appendix A in S1 File. Single-end sequencing of 150 bp reads was conducted on an Illumina MiSeq sequencing system in the Genomics Core Facility at the Pompeu Fabra University.

### Processing of sequencing output and identification of query sequences

Raw sequencing output was delivered in the form of de-multiplexed FASTQ files. We used the fastx_trimmer, part of the FASTX Toolkit (http://hannonlab.cshl.edu/fastx_toolkit/), to trim the output sequences and convert them to FASTA format. Specifically, we cut off the primer sequence and removed all reads that were shorter than 124 bp after trimming. We then used SEED [46] to cluster all reads with a divergence of 3 bp or less. After that, we discarded all clusters that consisted of 10 reads or less [9]. To eliminate chimera sequences we employed the uchime_denovo command of the USEARCH software [47]. Since all samples were amplified together and chimeras between amplicons of different samples could have been generated, we performed a joint analysis of multiplexed samples. We therefore joined all individual FASTA files before running USEARCH and separated them again afterwards. The number of reads of each cluster was associated to the cluster name, thus allowing a quantification of prey items in each sample.

For the identification of the query sequences in a tree-based approach, we first downloaded sequences of potential prey species of the Pyrenean desman from the BOLD database [13]. Specifically, we included aquatic macroinvertebrates that had been reported as prey species for the Pyrenean desman in previous studies [31–33]. Although terrestrial groups were noted in some studies, particularly in the most recent genetic studies [37], they were left out from this part of the analysis, as they would have led to an extremely large reference tree. For selecting the species, we took advantage of the "freshwaterecology" database, which compiles European taxa of aquatic organisms [22]. Species from the following taxa were selected: Ephemeroptera, Plecoptera, Trichoptera, Odonata, Neuropteroida (Megaloptera + Neuroptera*), Chironomidae, Diptera* excl. Chironomidae, Coleoptera*, Crustacea*, Bivalvia*, Gastropoda*, Annelida* excl. Hirudinea, Hirudinea. In taxa marked with an asterisk (*), only families with aquatic life stages were selected. Often, no sequences from the Iberian Peninsula were available. We therefore used sequences from other regions of Europe or, if none or too few were available, from anywhere in the world. We kept only sequences identified to species level (S1 Table). We aligned the sequences with MAFFT 7.130 [48,49]. Then, we used RAxML 8.2.9 [50] to reconstruct a maximum-likelihood tree of the reference dataset. After inspecting the output tree we manually pruned any sequences that were placed on exceptionally long branches or in the wrong higher-level taxa, as these were assumed to represent identification or sequencing errors, leaving 2130 reference sequences. Finally, we repeated the RAxML run with the pruned dataset. The final reference tree from these sequences is provided in S2 File.

We concatenated the reference dataset with the pre-processed sequences of all fecal samples and aligned the entire dataset with MAFFT [48,49]. We then used the evolutionary placement algorithm (EPA) [51] implemented in RAxML with default thresholds to place the query sequences in the reference dataset, using the previously reconstructed tree as guide tree. Information about mapped sequences and closest branches in the tree was saved in form of jplace output files [52] and subsequently parsed using the Genesis toolkit (http://genesis-lib.org/). We extracted the first (best) placement of each cluster, i.e., the species name of the closest leaf, according to its likelihood, but also recorded the species names of all other placements. We also recorded cases in which a sample was placed in more than one order (S2 Table), but only used the best result for further analyses.

As already stated, our reference dataset consisted exclusively of freshwater invertebrates that were considered potential prey items of the Pyrenean desman. However, we expected that PCR amplification would also yield sequences from organisms that were not part of the diet but that could be present in excrements, such as fungi and bacteria, or other ingested species not included in our reference tree. The tree-based identification would still assign such sequences to their most closely related reference sequence, leading to misidentification. Such misidentified sequences can be detected because the genetic divergence to their nearest neighbor is substantially larger than in correctly identified target sequences. Consequently, we searched for outlier branch lengths of the query sequences. For this purpose, we reconstructed the distribution of terminal branch lengths of the reference tree and identified the 99.9% quantile, which was 0.773 substitutions per position. For comparison we also applied a 99% quantile for outlier detection, which corresponded to 0.427 substitutions/position. All query sequences with branch lengths (‘pendant length’ in the jplace file) shorter than this threshold were considered identified. By using this threshold rather than the likelihood weight ratio we gave priority to the identification of clusters corresponding to some target taxonomic group, that is, we accepted an identification as long as the pendant length is short, which ensures that it belongs to the tree, even if the exact placement is uncertain. However, the species name assigned may be incorrect if there is uncertainty among several alternative placements or the true species is not represented in the reference database, which may happen often. We therefore treat our identification results as operational taxonomical units (OTUs) rather than species.

In addition to the tree-based identification, we used BLAST search [39] against the GenBank and BOLD databases for every cluster (i.e., one sequence per different OTU and excrement). We performed the searches with these sequences and noted the top result for each query sequence and database. We then compared these results with the result of the tree-based identification and assigned a score (ID score) from 0 to 18 as the sum of all three pairwise comparisons according to the following system: 6 = same species, 5 = same genus, 3 = same (insect) order, and 1 = same phylum.

Finally, we calculated the Shannon (H' [53]) diversity index for the identified species of each sample, using the number of reads associated to each OTU as surrogate of abundance. We conducted these analyses specifically for the samples of Zamora because they represented the largest assemblage of samples from the same area. There, we compared species richness between the two main rivers (Tera and Tuela) and measured beta diversity using the Jaccard index *J* [54,55] to quantify the differentiation between their biological communities, as characterized from the desman feces.

The pipeline protocol with all commands and custom Perl scripts produced to process data at different steps is given in Appendices B and C in S1 File.

## Results

After sequencing the library, we discarded six of the original 47 samples because they yielded less than 20 reads. All further analyses were conducted with the 41 successful samples (Table 1). Their sequencing yielded an average of 119,863 (with a range from 9,309 to 267,589) reads per sample. The clustering algorithm rendered 4,923 sequences. After eliminating chimera sequences 4,139 sequences remained and, after applying the RaxML EPA algorithm of tree-based identification and filtering for long branches, we obtained 2,374 identified target sequences across all samples. An overview of some characteristics of the samples is given in Table 1. A full list of all identification results is given in S2 Table.

A mean of 42% of reads per sample (< 1% - 90%) represented target reads, i.e., they were correctly identified as sequences of target organisms. Samples were found to contain an average of 15.0 (2–36) OTUs, and mean H' was 0.74 (0.02–1.76).

**Fig 1:**
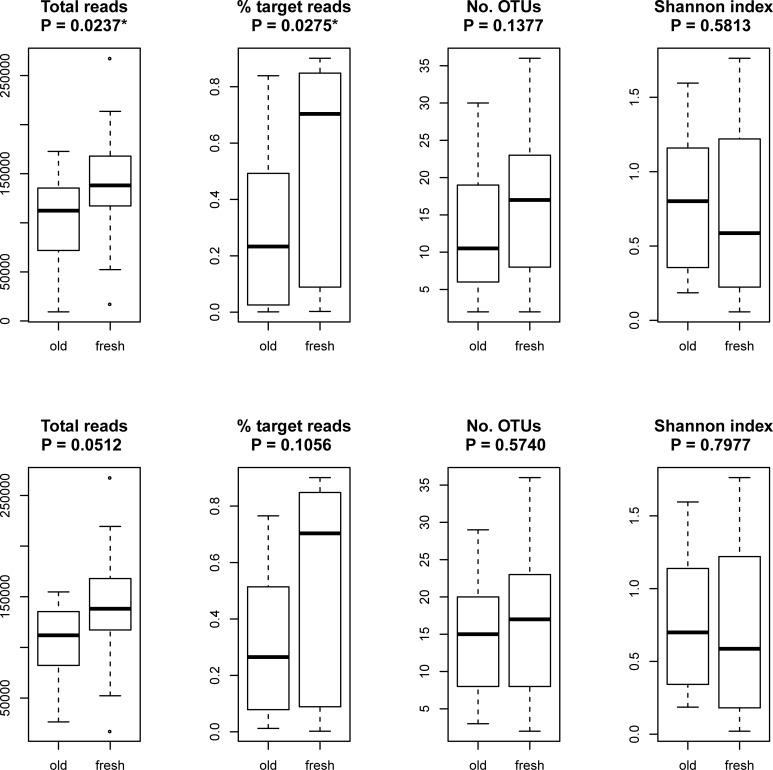
Box plots of some characteristics of old vs. new samples: Total reads per sample, percent of target reads, OTUs, and Shannon diversity index. Values marked with an asterisk (*) have a significantly higher median in fresh samples (P ≤ 0.05). Top row: all samples; bottom row: only samples from Zamora.

The comparison of fresh and old samples (Fig 1) showed that fresh samples yielded on average significantly more reads per sample (142,814 vs. 100,041; P = 0.0237*) and had a significantly higher proportion of target reads (56% vs. 29%; P = 0.0275*). The mean number of OTUs per sample was higher in fresh samples (17.4 vs. 12.9), whereas mean H' was higher in old samples (0.69 vs. 0.79), but neither of these differences was significant. A similar situation was found when using samples from Zamora alone (Fig 1).

**Fig 2:**
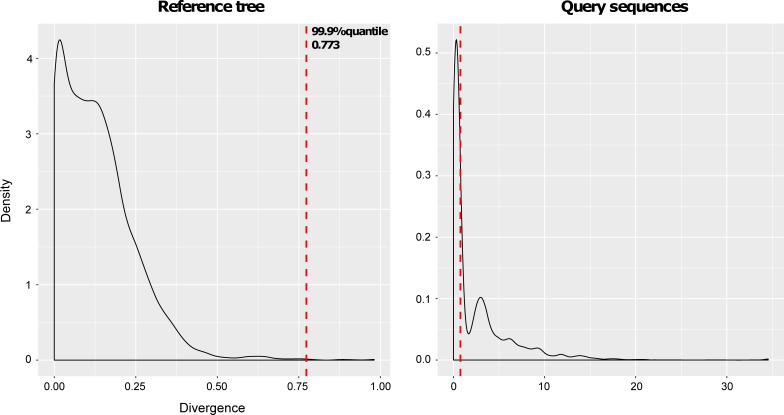
Distribution of divergence (branch lengths) of terminal branches in all sequences of the reference tree and in query sequences. The threshold branch length marking the 99.9% quantile of all reference sequences, shown in both plots with a vertical red line, is 0.773. Query sequences with branch lengths above the threshold are considered misidentified.

We compared the results of the tree-based identification to the BLAST results against the GenBank and BOLD databases. For this, we used a reduced dataset in which we selected one sequence per OTU and excrement, leaving 1,183 sequences (S2 Table). Of these, 614 were identified with an adequate branch length using the phylogenetic method and a 99.9% threshold of terminal branch lengths for outlier detection (Fig 2). By using a more stringent threshold (99%; see Methods), 513 sequences could be identified. In contrast, 218 sequences were detected with BOLD and 220 with GenBank, using an identity score equal to or greater than 97% [13]. If the minimum identity score was 98% [37], identified sequences would be 139 and 161 for BOLD and GenBank, respectively.

Using the ID score we devised, we found that this ID decreased with increasing minimum genetic divergence between any sequence in a cluster of query sequences and the closest reference sequence in the tree (Fig D in S1 File). This means that the three identification methods produced more similar results when query sequences were genetically more similar to a reference sequence. Overall, 56 sequences (4.73% of all sequences compared) produced identical identification to species level in all three methods.

The overall taxonomic composition of the samples is given in Table 2. The total number of clusters identified as different target OTUs was 224, included in 140 genera. Among the target groups of freshwater invertebrates, Ephemeroptera, Plecoptera, Trichoptera, Neuropteroida, Chironomidae, Diptera excl. Chironomidae, Coleoptera, Crustacea, and Annelida excl. Hirudinea were detected in our sequencing run, but not Odonata, Gastropoda, Bivalvia, and Hirudinea. Among them were 37 OTUs of Ephemeroptera (represented by 211 different sequences), 46 Diptera excl. Chironomidae (137 sequences), and 66 Chironomidae (126 sequences). These groups also represented the largest proportion of all target reads, with 60% in Ephemeroptera, 11% in Diptera excl. Chironomidae, and 19% in Chironomidae. A similar situation was revealed when we analyzed the top represented species with total read number and number of samples in which the species is present (Table 3). The top ten OTUs with most reads comprised OTUs of Ephemeroptera, one of Diptera excl. Chironomidae, and three of Chironomidae. The top ten of OTUs represented in most samples comprised six OTUs of Ephemeroptera, one of Diptera excl. Chironomidae, and two of Chironomidae. However, at the species level there are important differences between both rankings. For example, *Takobia albinatii* appears in 11 samples, being the 6th most represented species, but the total read number is low, ranking it in the 24th place. In the other extreme, *Paratanytarsus dissimilis* is ranked in the 6th position according to read number but it is present in only a single sample and thus ranked in position 16.

**Table 2:** Taxonomic composition of the samples, grouped by insect orders and the non-insect groups Annelida, and Crustacea (paraphyletic). Values given are absolute number of species of a group detected in a sample, with proportional number of reads representing this group in brackets.

	Chironomidae	Coleoptera	Diptera (excl. Chironomidae)	Ephemeroptera	Neuropteroida	Plecoptera	Trichoptera	Annelida	Crustacea
**Total**	66 (19%)	31 (1%)	46 (11%)	37 (60%)	3 (0%)	14 (0%)	22 (8%)	3 (0%)	2 (0%)
**BC0023**	21 (100%)	4 (0%)	6 (0%)	2 (0%)	1 (0%)	1 (0%)	1 (0%)	0 (0%)	0 (0%)
**BC0024**	0 (0%)	1 (0%)	1 (0%)	6 (100%)	0 (0%)	0 (0%)	0 (0%)	0 (0%)	0 (0%)
**BC0028**	4 (0%)	2 (2%)	1 (0%)	9 (98%)	0 (0%)	0 (0%)	0 (0%)	0 (0%)	0 (0%)
**BC0041**	0 (0%)	0 (0%)	2 (11%)	1 (38%)	0 (0%)	0 (0%)	2 (50%)	0 (0%)	0 (0%)
**BC0061**	1 (0%)	0 (0%)	2 (0%)	11 (100%)	0 (0%)	1 (0%)	2 (0%)	0 (0%)	0 (0%)
**BC0240**	5 (33%)	2 (16%)	2 (0%)	1 (49%)	0 (0%)	2 (1%)	0 (0%)	2 (1%)	0 (0%)
**BC0293**	21 (98%)	4 (0%)	8 (2%)	2 (0%)	0 (0%)	1 (0%)	0 (0%)	0 (0%)	0 (0%)
**BC0648**	0 (0%)	0 (0%)	1 (5%)	5 (95%)	0 (0%)	0 (0%)	0 (0%)	0 (0%)	0 (0%)
**BC0780**	0 (0%)	0 (0%)	2 (16%)	8 (84%)	0 (0%)	0 (0%)	0 (0%)	0 (0%)	0 (0%)
**BC0834**	5 (22%)	1 (24%)	3 (0%)	2 (4%)	0 (0%)	1 (0%)	3 (50%)	0 (0%)	0 (0%)
**BC0849**	3 (4%)	2 (6%)	5 (4%)	7 (46%)	0 (0%)	2 (40%)	1 (1%)	0 (0%)	0 (0%)
**BC0857**	3 (71%)	0 (0%)	2 (20%)	2 (7%)	0 (0%)	0 (0%)	1 (2%)	0 (0%)	0 (0%)
**BC0861**	1 (0%)	2 (35%)	4 (63%)	2 (1%)	0 (0%)	0 (0%)	0 (0%)	0 (0%)	0 (0%)
**BC0868**	0 (0%)	1 (0%)	4 (0%)	14 (100%)	0 (0%)	5 (0%)	0 (0%)	0 (0%)	0 (0%)
**BC0873**	2 (1%)	0 (0%)	1 (0%)	2 (1%)	0 (0%)	0 (0%)	3 (98%)	0 (0%)	0 (0%)
**BC0877**	1 (0%)	0 (0%)	1 (0%)	4 (10%)	0 (0%)	0 (0%)	8 (89%)	0 (0%)	0 (0%)
**BC0899**	1 (0%)	2 (0%)	12 (100%)	2 (0%)	0 (0%)	0 (0%)	0 (0%)	0 (0%)	0 (0%)
**BC0917**	2 (0%)	0 (0%)	1 (0%)	6 (24%)	0 (0%)	4 (0%)	13 (76%)	0 (0%)	0 (0%)
**BC0939**	2 (0%)	2 (0%)	13 (99%)	2 (1%)	0 (0%)	0 (0%)	1 (0%)	1 (0%)	0 (0%)
**BC0943**	0 (0%)	0 (0%)	2 (94%)	3 (6%)	0 (0%)	0 (0%)	0 (0%)	0 (0%)	0 (0%)
**BC0974**	4 (0%)	3 (1%)	15 (97%)	5 (2%)	0 (0%)	0 (0%)	2 (0%)	0 (0%)	0 (0%)
**BC0981**	7 (64%)	2 (20%)	2 (0%)	5 (15%)	1 (0%)	0 (0%)	2 (0%)	0 (0%)	0 (0%)
**BC1035**	0 (0%)	1 (22%)	1 (7%)	4 (66%)	0 (0%)	1 (5%)	0 (0%)	0 (0%)	0 (0%)
**BC1041**	2 (0%)	2 (3%)	2 (0%)	12 (95%)	0 (0%)	2 (2%)	0 (0%)	0 (0%)	0 (0%)
**BC1059**	0 (0%)	3 (0%)	3 (0%)	11 (100%)	0 (0%)	0 (0%)	0 (0%)	0 (0%)	0 (0%)
**BC1062**	0 (0%)	0 (0%)	1 (76%)	1 (24%)	0 (0%)	0 (0%)	0 (0%)	0 (0%)	0 (0%)
**BC1101**	2 (0%)	2 (0%)	1 (0%)	11 (100%)	0 (0%)	0 (0%)	1 (0%)	0 (0%)	0 (0%)
**BC1108**	4 (0%)	0 (0%)	0 (0%)	14 (100%)	2 (0%)	0 (0%)	1 (0%)	0 (0%)	0 (0%)
**BC1123**	1 (0%)	1 (0%)	2 (0%)	12 (100%)	0 (0%)	0 (0%)	0 (0%)	0 (0%)	0 (0%)
**BC1144**	2 (0%)	2 (0%)	10 (91%)	4 (3%)	0 (0%)	2 (5%)	2 (0%)	0 (0%)	1 (0%)
**BC1150**	4 (0%)	1 (0%)	1 (0%)	6 (99%)	1 (0%)	3 (0%)	1 (0%)	0 (0%)	0 (0%)
**BC1154**	3 (9%)	0 (0%)	1 (9%)	2 (82%)	0 (0%)	0 (0%)	0 (0%)	0 (0%)	0 (0%)
**BC1243**	2 (0%)	5 (0%)	4 (0%)	14 (99%)	0 (0%)	4 (1%)	0 (0%)	0 (0%)	0 (0%)
**C1131**	18 (99%)	4 (0%)	2 (0%)	2 (0%)	1 (0%)	1 (0%)	1 (0%)	0 (0%)	1 (0%)
**C1654**	0 (0%)	0 (0%)	4 (26%)	6 (64%)	0 (0%)	1 (11%)	0 (0%)	0 (0%)	0 (0%)
**C1661**	0 (0%)	0 (0%)	2 (3%)	4 (96%)	0 (0%)	0 (0%)	1 (0%)	0 (0%)	0 (0%)
**C1671**	3 (23%)	0 (0%)	0 (0%)	2 (78%)	0 (0%)	0 (0%)	0 (0%)	0 (0%)	0 (0%)
**C1796**	0 (0%)	0 (0%)	1 (26%)	1 (74%)	0 (0%)	0 (0%)	0 (0%)	0 (0%)	0 (0%)
**C3855**	0 (0%)	0 (0%)	1 (2%)	2 (98%)	0 (0%)	0 (0%)	0 (0%)	0 (0%)	0 (0%)
**C4323**	2 (21%)	0 (0%)	1 (30%)	1 (49%)	0 (0%)	0 (0%)	0 (0%)	0 (0%)	0 (0%)
**OHGC001**	0 (0%)	2 (0%)	10 (99%)	1 (0%)	0 (0%)	0 (0%)	1 (0%)	0 (0%)	0 (0%)

**Table 3:** The OTUs registered with most sequencing reads and detected in most samples.

Species (group)	Sum reads	No. samples	Rank reads	Rank samples
*Baetis lutheri* (Ephemeroptera)	807352	34	1	1
*Ecdyonurus venosus* (Ephemeroptera)	314287	28	2	3
*Dixella* sp. (Diptera)	204175	31	3	2
*Rheocricotopus atripes* (Chironomidae)	158227	11	4	6
*Baetis fuscatus* (Ephemeroptera)	110404	17	5	4
*Paratanytarsus dissimilis* (Chironomidae)	92595	1	6	16
*Allogamus mortoni* (Trichoptera)	91546	9	7	8
*Macropelopia notata* (Chironomidae)	74331	13	8	5
*Philopotamus ludificatus* (Trichoptera)	62941	6	9	11
*Rhithrogena semicolorata* (Ephemeroptera)	50159	10	10	7
*Habroleptoides* sp. SC2014 (Ephemeroptera)	37653	13	13	5
*Takobia albinatii* (Ephemeroptera)	10386	11	24	6

Non-target organisms identified by BLAST with an identity of at least 97% with either BOLD or GenBank (Table E in S1 File) comprised Rotifera (found in 23 samples), Oomycota (20 samples), Algae (Rhodophyta and Chlorophyta, 13 samples), various Protists (13 samples), Hemiptera (13 samples), Ascomycota (12 samples), Collembola + Protura (eight samples), Acari (eight samples), Hymenoptera and Nematoda (each three samples), Cnidaria (two samples), and others. All of these groups were detected by BOLD and GenBank BLAST in old as well as fresh samples. The notable exception was the genus Saprolegnia (Oomycota), which was exclusively detected in old fecal samples. No sequence of the Pyrenean desman was retrieved from any sample.

The analysis of the Tuela and Tera river systems showed richness of 133 OTUs in Tuela and 138 in Tera, amounting to a total richness of 202 with 69 shared species (*J* = 0.34).

## Discussion

### Amplicon sequencing of fecal samples: Methodological issues

This study represents an attempt to use COI amplicon sequencing by NGS from fecal samples of a semi-aquatic mammal as a means to study its diet. The analysis of fecal samples for diet studies involves some important problems that may potentially bias the results. First, PCR based on a single set of primers may fail to amplify all diet species. Second, samples collected in rivers may have been exposed to contamination with eDNA from the water or from coprophagous organisms for days or weeks. Third, DNA degradation during digestion or exposure to the environment may prevent the amplification of an important part of the diet species. Fourth, target (i.e., food) sequences are usually mixed with a large amount of intestinal organisms, such as bacteria and protozoans. Fifth, the identification of prey species that span all metazoans with mtDNA is prone to phylogenetic artifacts that may arise in the comparison of highly divergent sequences. Although next-generation sequencing techniques have opened a promising path to obtain massive information on the diet composition of species, these methodological problems need to be addressed before proceeding to wide-range surveys of diet.

One way to increase barcoding success is using highly specific primers [56]. However, since we targeted organisms from a wide range of animal taxa, we used a set of universal primers that amplified different metazoan species. We considered a pre-amplification step in our protocol helpful to increase the number of reads corresponding to target taxa (i.e., animals), but this nested PCR approach may have also increased the specificity of the amplification. It is therefore possible that we failed to sequence an unknown amount of diet species. As shown in Morinière et al. [9], many COI primers have highly variable amplification success even among members of their target groups, i.e., a primer pair specifically developed for reptiles may fail to amplify some species even if congeners are successfully amplified [57]. Therefore, future studies should use primer cocktails or pool the amplification products of more than one primer pair to improve the amplification success of the target group.

The use of excrements exclusively collected in rivers to study the diet of the Pyrenean desman would have been highly problematic because we would not have been able to demonstrate that many sequences, especially those present at low levels, come from ingested food and not from eDNA. In the same manner, excrements collected this way would have made it difficult for us to determine if many prey sequences in excrements would be lost due to degradation. The availability in our study of fresh excrements from captured specimens, where eDNA contamination was highly improbable and DNA degradation was stopped by immediate storage of the excrements in ethanol, allowed us addressing the problems with eDNA contamination and DNA degradation at the same time. Interestingly, fresh samples produced significantly more target reads and a higher proportion of target reads with respect to total reads; they also produced more OTUs, albeit not significantly. Therefore, some degradation seems to have occurred in old samples, as expected, but the generally high number of reads obtained from them seems to be sufficient to capture a significant number of OTUs.

**Fig 3:**
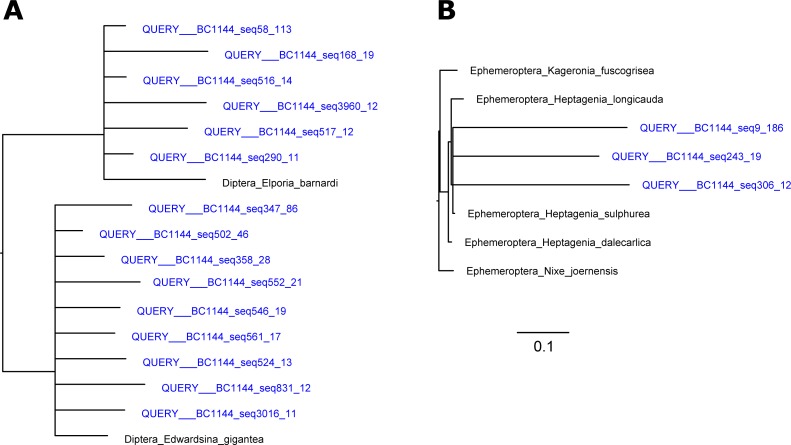
A subset of the reference tree for visualization of the identification of query sequences. Reference sequences in black, query sequences with number of sample, number of ID, number of sequence, and number of assembled reads in blue. A: In many cases, query sequences cluster with the closest reference sequence with branch lengths shorter than interspecific divergences. B: Query sequences with very long branches separating them from reference sequence are discarded as likely misidentifications.

The wide range of potential metazoan species that can be present in the diet of the Pyrenean desman means that highly divergent sequences have to be compared in the identification process. The identification of these sequences by BLAST has the advantage of using large databases for the comparison and the possibility of obtaining an exact species identification. However, this method may be problematic because it does not take into account evolutionary rate variation. For example, potential species with relatively high rates may be undetected by BLAST in favor of other, more slowly evolving species [40,41]. This problem may be exacerbated when the identification is performed at higher taxonomic levels [58]. Some of these problems can partly be amended by tree-based identification methods using a tree of *a priori* defined target species. The raw results will then assign the closest target species in the tree, taking into account all phylogenetic information contained in the sequences [51,59]. We therefore tested the efficiency of tree-based identification of our sequences by comparing the results of this approach to those of BLAST identification. Typically, non-target sequences are represented by conspicuously long branches (Fig 3). We chose the 99.9 percentile of the distribution of terminal branch lengths of reference sequences as a cutoff value for accepting tree-based identification results of query sequences as correct. The comparison with BLAST identification results showed that query sequences below this threshold were generally identified as target species also in the BLAST search, whereas BLAST results for sequences above the threshold were often non-target species. As expected, the identification results tended to be more convergent in query sequences that were more similar to a known reference sequence (Fig D in S1 File).

While we have established a rigorous protocol for processing the sequences, the actual species identification can only be achieved by having databases with all species from the studied area, which at this moment is not available. For example, only 206 of the 638 (32%) species of Ephemeroptera, Plecoptera, and Trichoptera listed for Spain by the freshwaterecology database [22] were available on BOLD, as of June 2017. We therefore treat our identification results as OTUs rather than species.

### Species identification and quantitative composition of diet data in feces

Comparing the Shannon diversity indices (H') based on read numbers of each OTU in the samples shows clear differences in diversity between samples (Table 1). In some samples with low H', nearly 100% of the reads represent a single target OTU, whereas in samples with high H' the reads are more evenly distributed over several target OTUs (Table 1). The number of reads associated with each OTU might be suggested to represent the actual amount of biomass of a prey species consumed by a desman individual. However, most studies argue that any correlation between biomass and reads should be treated with caution [60,61], at least without calibration by a quantitative mockup sample [62]. This is probably equally true for our samples. There are many other factors influencing the sequencing success and the resulting number of reads in amplicon sequencing in general, foremost among them the specific degree of fitting of primers and protocol used and the specific amount and quality of DNA extracted from different types of tissue (mostly different between phyla; Pawluczyk et al. 2015; Morinière et al. 2016). In fecal samples, the time passed from ingestion of a prey item to defecation, the specific resistance of the tissue to digestion, and the time and place of exposition after defecation may be even more influential on the results [63,64]. Nevertheless, very clear cases of, e.g., ~99% of reads in one OTU (fresh sample BC0024) vs. ~97% of reads more or less evenly distributed among five prey OTUs (fresh sample BC0240) may be indicative of different feeding strategies. Thus, a quantitative analysis of read numbers with a larger sample size may allow studying such feeding strategies in different areas or at different times of the year. It may also be possible to study if the desman feeds on all available macroinvertebrates present in the river or if there is prey selection. Clearly, further studies are necessary to gain more insight into these questions.

Sequences with a long branch in the tree-based identification method were unlikely to come from the target freshwater organisms. In these cases, and despite the problems mentioned above, we could only use the species identification provided by the BLAST search. As expected, these sequences were often identified as organisms possibly involved in decomposition. Most commonly represented were Rotifera and aquatic fungi (mostly Oomycota), but also unicellular organisms. These may be involved in decomposition or represent contamination by eDNA. Protura and Collembola possibly contaminated samples by feeding on the fungi growing on the feces. Another small proportion of non-target sequences was identified as terrestrial insects (Hemiptera and Hymenoptera). These were possibly ingested after falling into the water, although it cannot be excluded that the Pyrenean desman also seeks for them in riverbanks (see below). Cnidarian reads may be a result of contamination by eDNA. The only group detected exclusively in old samples was fungi of the genus *Saprolegnia* (Oomycota), which suggests that these organisms colonize feces after defecation.

### Diet of the Pyrenean desman

Overall, our results are similar to those of previous studies on the diet of the Pyrenean desman [31–33,36–38]. All studies found high amounts of Ephemeroptera, Plecoptera, and Trichoptera. Castién & Gosálbez [32], Fernández-Salvador et al. [33], and Biffi et al. [37] also found substantial numbers of Diptera, including Chironomidae. Like our study, Castién & Gosálbez [32], Santamarina [31], Fernández-Salvador et al. [33], and Biffi et al. [37] found small amounts of Coleoptera and Annelida (in the case of Fernández-Salvador et al. [33] these were Hirudinea). Castién & Gosálbez [32] and Fernández-Salvador et al. [33] were the only ones to find Gastropoda, and eggs of trouts (*Salmo trutta*) were exclusively detected in Castién & Gosálbez [32]. Biffi et al. [37] also noted Crustacea (Amphipoda). Crustaceans were also detected in Santamarina [31] and Fernández-Salvador et al. [33], and Neuropteroida were represented in Santamarina [31], Fernández-Salvador et al. [33], Biffi et al. [37] and some of our samples. Furthermore, Odonata was detected in some studies in small quantities despite the known presence of this group in the studied regions, although these species are not as abundant as other invertebrates, probably explaining their scarcity in desman excrements.

A substantial proportion of excrements with terrestrial species such as Orthoptera, Hemiptera and Lepidoptera, among others, was reported in the genetic study of Biffi et al. [37]. However, since Biffi et al. [37] only used frequencies of occurrence and did not associate read numbers to each species, these molecular data do not allow a quantitative assessment of the prey consumed, as already suggested [38]. Notably, studies based on morphological examination of gut contents also found terrestrial organisms, but only at much lower proportions [31–33]. As we did not include terrestrial species in our reference dataset, we could not detect such species using the tree-based identification. However, our BOLD and GenBank BLAST searches allowed the detection of terrestrial OTUs in our samples. When we used the 97% threshold in BOLD and GenBank, we did not find any OTU belonging to either Orthoptera or Lepidoptera. On the other hand, we detected one species of terrestrial Hemiptera (*Kleidocerys ericae*) in 12 of our samples. Conceivably, these true bugs might be consumed by *Galemys* during aquatic (after falling to the river) or terrestrial foraging. However, the regular occurrence of this single terrestrial species, versus non-detection of any other potential terrestrial prey items, suggests that the detection of this taxon may be due to spurious COI similarity with an aquatic organism from a close taxonomic group. Other sequences identified by BLAST that might be terrestrial were a minority in our samples. In contrast to Biffi et al. [37], but in agreement with all previous studies, the results based on our samples and methods suggest that terrestrial prey items make up a potentially detectable, but minor part of the diet of the Pyrenean desman. However, it cannot be discarded that in some specific habitats the accumulation of terrestrial species may be higher and that the desman could profit from this resource, as it may be the case in the previous genetic studies [37], but a quantitative analysis of read numbers in more areas of the distribution should be performed in order to know with certainty the real impact of this resource on the diet of the Pyrenean desman.

The comparison of our overall results with previous genetic studies shows considerable overlap, but also some remarkable differences. Biffi et al. [37] identified a mean of 5.8 genera per sample. In comparison, we identified a mean of 15 clusters per sample. The phylogenic method we used, which allowed the detection of a higher proportion of sequences, together with the higher sequencing depth of our study (ca. 10,000 sequences per sample versus ca. 7,000 in Biffi et al. [37]) may explain these contrasting results. Nevertheless, in comparison with these studies, which used a one-step PCR, our laboratory method involved two nested PCR steps, which allowed the successful amplification of a high number of excrements but may have caused some species to fail amplification due to increased specificity. Future work should address this potential issue because, in fact, the diversity of prey species in the desman diet could be even higher. However, our results suggest that metabarcoding with deep sequencing, the use of a tree-based identification and the quantification of read number has the potential of providing a comprehensive representation of prey diversity, given that a reliable reference database for species-level identification is available.

Comparing the Tuela and Tera river systems showed that, despite geographic proximity of the systems, and despite similar species richness, only a relatively small proportion of all OTUs was detected in both systems (*J* = 0.34). All major organism groups were detected in both systems, with the exception of the few samples with Annelida (only Tuela) and Crustacea (only Tera). As samples from both rivers were collected from late spring to late fall, we do not expect seasonal variation in prey preference of the desman to be the reason for this difference. However, it is likely that further sampling is necessary to achieve saturation in prey detection, highlighting the importance of using metabarcoding approaches that recover as many OTUs as possible to understand the variability of the diet of the Pyrenean desman in different river systems and habitats.

### Conclusions, outlook, and applications

In this paper, we attempted to provide and test a pipeline for metabarcoding of fecal samples developed to study the diet of the Pyrenean desman, but valid for any other species. It should be taken into account that the primary objective of this study was to test our metabarcoding pipeline. Any more comprehensive characterization of the diet of the Pyrenean desman will require a larger sample size and year-round sampling, as was done in some previous studies. Despite these limitations, our results suggest that feces of the Pyrenean desman found in the habitat of the species, the only form of obtaining this material without capturing individuals, are in principle suitable for diet studies in combination with our pipeline. However, these results also indicate that fresh samples may yield more reads, a higher proportion of target reads, and potentially more OTUs and therefore the extra effort of preferentially collecting the freshest samples from rivers may help to ensure more robust results on the diet of the Pyrenean desman.

Our pipeline provides access to a wide range of potential applications. First of all, it allows studying the diet of species using non-invasive samples, which is crucial for endangered species like the Pyrenean desman. Rigorous quantitative knowledge on the feeding habits of such species is a fundamental step to develop conservation programs, including reintroduction plans if they become necessary, since only rivers with abundant and adequate food for the species should be considered for potential reintroductions. If enough data on diet is available, this will also allow the comparison between different conspecific populations and detect shifts in diet depending on the presence or absence of competitors, predators or anthropogenic modification of the habitat. In addition, the comparison of samples collected in different seasons may provide information on diet variability along the year. Furthermore, the data gained from studies of a semi-aquatic mammal like the Pyrenean desman may be used to assess the communities of freshwater organisms. While there will be a bias based on the dietary preference of this insectivore, this approach may grant relatively easy access to a large amount of river biodiversity data because the desman, or other aquatic or semi-aquatic insectivores, 'condense' the original sample volume through their foraging. This may be particularly useful in lotic water, where the sampling of eDNA may be difficult [65]. We therefore hope that our results will be an incentive for a wider extension of metabarcoding in ecological analyses and monitoring.

## Supporting information

S1 File Supporting text and data. Protocol for amplicon library preparation, bioinformatical pipeline for the analysis of amplicon sequencing results, perl scripts used in the bioinformatical pipeline, plot of genetic divergence and ID score including only target sequences, summary of the results of BOLD and GenBank BLAST identification of non-target reads.
(PDF)

S2 File Reference tree file for tree-based identification. The tree file was generated based on data from S1 Table.
(TRE)

S1 Table Table with reference sequences and metadata used to produce the reference tree. All data is from the BOLD database.
(XLSX)

S2 Table Results of tree-based vs. BOLD and GenBank BLAST identification. (XLSX)
