# Supplementary material for: A pipeline for metabarcoding and diet analysis from fecal samples developed for a small semi-aquatic mammal
Source: PLoS One. 2018 Aug 14;13(8):e0201763. doi: 10.1371/journal.pone.0201763 (PMC6091967; doi:10.1371/journal.pone.0201763)
Supplement: S1 File — Protocol for amplicon library preparation, bioinformatical pipeline for the analysis of amplicon sequencing results, perl scripts used in the bioinformatical pipeline, plot of genetic divergence and ID score including only target sequences, summary of the results of BOLD and GenBank BLAST identification of non-target reads. (PDF) [file pone.0201763.s001.pdf]

Supporting information:

A pipeline for metabarcoding and diet analysis from fecal samples developed for a small semi-aquatic mammal

Oliver Hawlitschek[1], Angel Fernández-González[2], Alfonso Balmori-de la Puente[1], Jose Castresana[1]

[1] Institut de Biologia Evolutiva (CSIC-Universitat Pompeu Fabra), Passeig Maritim de la Barceloneta 37, 08003 Barcelona, Spain

[2] Biosfera Consultoría Medioambiental S.L., Calle Candamo 5, 33012 Oviedo, Spain

S1 File

Contents:

A Protocol for amplicon library preparation.

B Bioinformatical pipeline for the analysis of amplicon sequencing results.

C Perl scripts used in the bioinformatical pipeline.

D Plot of genetic divergence and ID score, including only target sequences.

E Summary of the results of BOLD and GenBank blast identification of non-target reads.

A Protocol for amplicon library preparation.

Primers: Order primers ready with adapters. Primers are based on Illumina Nextera XT Kit.

1. Minibarcode primers (specific for the COI minibarcode region) with overhang sequences (part 1 of the Illumina adapters) for Amplicon PCR. Length of the minibarcode region: 130 bp

5'-TCGTCGGCAGCGTCAGATGTGTATAAGAGACAG-[minibarcode-primer-F]-3'
5'-GTCTCGTGGGCTCGGAGATGTGTATAAGAGACAG-[minibarcode-primer-R]-3'

Uni-MinibarF1: 5'-TCCACTAATCACAARGATATTGGTAC-3'
Uni-MinibarR1: 5'-GAAAATCATAATGAAGGCATGAGC-3'

2. Index sequences (specific for each sample) and part 2 of the Illumina adapters for Index PCR.

5'-AATGATACGGCGACCACCGAGATCTACAC-[i5]-TCGTCGGCAGCGTC-3'
5'-CAAGCAGAAGACGGCATACGAGAT-[i7]-GTCTCGTGGGCTCGG-3'

Red / blue: Overlapping regions of the adapters.

Total length of fragments (adapters + primers + sequences): 316 bp.

Use Nextera XT Index 5 and 7 adapters. Index sequences Nextera XT:

I7 bases	I7 name	I5 bases	I5 name
TCGCCTTA	N701	TAGATCGC	[N/S/E]501
CTAGTACG	N702	CTCTCTAT	[N/S/E]502
TTCTGCCT	N703	TATCCTCT	[N/S/E]503
GCTCAGGA	N704	AGAGTAGA	[N/S/E]504
AGGAGTCC	N705	GTAAGGAG	[N/S/E]505
CATGCCTA	N706	ACTGCATA	[N/S/E]506
GTAGAGAG	N707	AAGGAGTA	[N/S/E]507
CCTCTCTG	N708	CTAAGCCT	[N/S/E]508
AGCGTAGC	N709	GCGTAAGA	[N/S/E]517
CAGCCTCG	N710		
TGCCTCTT	N711		
TCCTCTAC	N712		

Pre-amplification using primers for full barcoding fragment:

- Prepare a mix (per sample):
15 µl H_2O MQ
5 µl GoTaq buffer HF 5x
0.5 µl 10 mM dNTPs
0.35 µl BSA
1.0 µl Pre-amplification Primer 1 (HCO; 2 µM final)
1.0 µl Pre-amplification Primer 2 (LCO; 2 µM final)
0.15 µl GoTaq Polymerase

- Add:
23 µl mix
2 µl template

- PCR:
Initial denaturation: 98 ºC (2 min)
35 cycles: 98 ºC (30 sec); 50 ºC (40 sec); 72 ºC (60 sec)
Final extension: 72 ºC (5 min)

- Purify, e.g., with Thermo Fisher ExoSAP-IT PCR purification kit.

Amplicon PCR

- Prepare a mix (per sample):
17 µl H_2O MQ
5 µl GoTaq buffer HF 5x
0.5 µl 10 mM dNTPs
0.35 µl BSA
0.5 µl Amplicon Primer 1 (2 µM final)
0.5 µl Amplicon Primer 2 (2 µM final)
0.15 µl GoTaq Polymerase

- Add:
24 µl mix
1 µl sample

- PCR:
Initial denaturation: 98 ºC (2 min)
35 cycles: 98 ºC (30 sec); 50 ºC (40 sec); 72 ºC (60 sec)
Final extension: 72 ºC (5 min)

- Purify, e.g., with Thermo Fisher ExoSAP-IT PCR purification kit.

Index PCR

- Prepare a mix (per sample):

17 µl H2O MQ
5 µl PHUSION buffer HF 5x
0.5 µl 10 mM dNTPs
0.35 µl BSA
0.5 µl Index Primer 1 (2 µM final)
0.5 µl Index Primer 2 (2 µM final)
0.15 µl PHUSION Polymerase

- Add:
24 µl mix
1 µl sample

- PCR:
Initial denaturation: 98 ºC (2 min)
Cycles: 98 ºC (30 sec); 50 ºC (40 sec); 72 ºC (60 sec)
Final extension: 72 ºC (5 min)
14 cycles (try more or less depending on band intensity)

- Check results on agarose gel.

Pool

- Prepare one tube (Falcon 15 ml sterile) with 125 µl of buffer PB per sample for purification. Add the entire PCR products of all indexed samples. Only pool bands that have strong intensity in the gel (or repeat if necessary).

- Run the pooled samples through a column of MinElute PCR Purification Kit in steps of ~750 µl; centrifuge each time after adding volume.

- Incubate 5 min and elute in 30 µl of EB.

Purification

- Measure rough DNA concentration in Nanodrop

- Run (5-18) µl of pool in an E-gel EX 2% (Invitrogen) for 13 min (a maximum of 400 ng DNA should be loaded).

- Cut the ~310-320 bp band containing the library.

- Purify the agarose slice with Qiaquick Gel Extraction Kit. Incubate for 5 min, elute in 30 µl EB and store in freezer.

B Bioinformatic pipeline for the analysis of amplicon sequencing results

Use de-multiplexed MiSeq sequencing data: *file.fastq* for each sample.

Use a fasta file with reference sequences for identification: *ref.fasta.*

And a tree generated from the reference data: *ref.tre.*

Software used:
FASTX toolkit
SEED
Usearch
MAFFT 7.130
RAxML 8.2.9
Genesis v0.12.1

Custom Perl scripts:
parseSEED.pl
ConcatSampNameSeq.pl
UchimePreParse.pl
UchimePostParse.pl
DeMultiUchim.pl
fasta2phy.pl
parse_jplace.pl

Trim sequences using FASTX toolkit

Command: fastx_trimmer -t 1 -m 150 -Q 33 -i *file.fastq* -o *filet.fastq*
-t 1: Remove last bp of sequences (in case 151 bp are delivered)
-m 150: Discard any sequence that is < 150 bp after trimming
-Q 33: specifies Phred score +33 (not by default)

Command: fastx_trimmer -f 27 -Q 33 -i *filet.fastq* -o *filet2.fastq*
-f 27: Remove first 26 bp of primer sequences (First base to keep: 27)

Use SEED for clustering sequences

Command: SEED --input *filet.fastq* --output *filets.fastq* –fast
Default maximum number of mismatches allowed is 3
-fast option can be used

Use the custom parseSEED.pl script to convert SEED output file to fasta format and associate number of sequences in this cluster to each cluster
Command: perl parseSEED.pl *filets.fastq* 10
Output file: *filets.fastqc.fas*
Header of every sequence: >(original number of first sequence in cluster)_(number of sequences in this cluster)
10: Minimum number of reads that clusters should have. Smaller clusters will be discarded.

Attach the sample name to every sequence in the fasta file using the custom ConcatSampNameSeq.pl script:
Command: perl ConcatSampNameSeq.pl *filets.fastqc.fas*
Output file: *filel.fas*

Eliminate chimera sequences using the uchime command from usearch

Concatenate all sample fasta files: This is necessary to detect also cross-sample chimeras.
Command: cat *filel.fas (1) … filel.fas (N) > allquery.fas*

Prepare uchime input format using the custom perl script UchimePreParse.pl:
Command: perl UchimePreParse.pl allquery.*fas*
Output file: *allquery.fasu1.fas*

Run uchime:
Command: usearch -uchime2_denovo *allquery.fasu1.fas* -uchimeout *allqueryChim* -nonchimeras *allqueryu.fas*
Produces:
allqueryChim: A file that lists detected chimera sequences.
allqueryu.fas: A fasta file from which chimera sequences have been eliminated.

Convert uchime output format to normal fasta using the custom perl script UchimePostParse.pl:

Command: perl UchimePostParse.pl *allqueryu.fas*
Output file: *allqueryu.fasu2.fas*

Demultiplex uchime output file and concatenate fasta files of reference with each sample separately

Use the custom DeMultiUchim.pl script to split the uchime output file into separate fasta files for all samples:
Command: perl DeMultiUchim.pl *allqueryu.fasu2.fas file*
Output file: *fileu.fas*

Concatenate *ref.fasta* and uchime output:
Command: cat *ref.fasta fileu.fas* > *filetscr.fas*

Align fasta files using MAFFT

Command: mafft.bat *filetscr.fas* > *filetscra.fastal*

Use the custom fasta2phy.pl script to convert files to phylip format:
Command: perl fasta2phy.pl *filetscr.fastal*
Output file: *filetscr.phy*

Run RAxML EPA to place the query sequences in the reference tree

Command: raxmlHPC -f v -p 886 -m GTRGAMMA -s *filetscr.phy* -t *ref.tre* -n *file*
-f v: Activates the EPA algorithm.

Run Genesis to parse the jplace files generated by RAxML EPA

Use the program placement_classification_table of the Genesis package to extract the closest species (there may be several):
Command: placement_classification_table *filetscr.jplace filetscr.jplace.out*

Parse the output of Genesis with the custom script parse_jplace.pl to get the first placement of each cluster according to its likelihood and recorded the order all other placements:
Command: parse_jplace.pl *filetscr.jplace.out*
Output file: *filetscr.jplace_2.out*

Concatenate all output files:
Command: cat *filetscr.jplace_2.out (1)* ... *filetscr.jplace_2.out (N)* > table2.txt

C Perl scripts used in the bioinformatical pipeline.

```
#!/usr/bin/perl

######### parseSEED.pl ###########
### A Perl script that parses SEED output files
### For joint metabarcoding analysis

if ( @ARGV < 2) {
	print "\nEnter a file from SEED and the minimum number of reads";
	print "\nto generate a fasta file with reads count\n\n";
	exit();
}

$file = $ARGV[0];

if ($file !~  m/\//) {$file = ".\/" . $file;} # si no se da el path
$position = rindex($file, "\/");
$path = substr($file, 0, $position+1);
$name = substr($file, $position+1);
$extposition = rindex($name, "\.");
$ext = substr($name, $extposition+1);
$namenoext = substr($name, 0, $extposition);
print ("\nFilename: $name\n");

open(FILE, $file)  or die "Can't open file";

$newfile = $file . "c.fas";

$x = -1;
while (<FILE>) {
	chomp;
	if (/CLID	SeqID/) {next;}
	if (!/\t/) {
		$x++;
		$count[$x] = 0;
		$seq[$x] = $_;
	}
	else {
		$count[$x]++;
	}
}

close(FILE);

$tot = $x+1;

print ("\nTotal number of sequences: $tot\n\n");
```

```
open (FILE2, "> $newfile");

for ($x = 0; $x < $tot; $x ++) {
		if ($count[$x] > $ARGV[1]) {
				print FILE2 (">seq$x\_$count[$x]\n");
				print FILE2 ("$seq[$x]\n");
		}
}

close(FILE2);
```

```
#!/usr/bin/perl
#use warnings;

######### ConcatSampNameSeq.pl ###########
### A Perl script that attaches the file name to sequence labels in a fasta file
### For joint metabarcoding analysis

### Call: ConcatSampNameSeq.pl <filename>
### Input: fasta file with query sequences, aligned

# Input filename
$file = $ARGV[0];

# Path is split into pieces to allow later renaming and saving of copies
if ($file !~  m/\//) {$file = ".\/" . $file;} # si no se da el path
$position = rindex($file, "\/");
$path = substr($file, 0, $position+1);
$name = substr($file, $position+1);
$extposition = rindex($name, "\.");
$ext = substr($name, $extposition+1);
$namenoext = substr($name, 0, $extposition);
print ("\nFilename: $name\n");

$possample = rindex($name, "ts");
$sample = substr($name, 0, $possample);
# $sample = substr($name, /[^0-9]+[0-9]+/);
print ("\nSample: $sample\n");
print ("\nName: $name\n");

# Open the input file
open(FILE, $file)  or die "Can't open file";

# Create an output file and open it
$newfile = $path . $sample . "l.fas";
open (FILE2, "> $newfile");

# Attache the filename to the beginning of each fasta name line
while (<FILE>) {
		chomp;
		if ($_ =~ /^>.*$/g) {
				$_ =~ s/>//g;
				print FILE2 (">", $sample, "\_", $_, "\n");
				}
		else {
				print FILE2 ($_, "\n");
				}
		}

# Close files
close(FILE);
```

```
close(FILE2);
```

```
#!/usr/bin/perl
#use warnings;

######### UchimePreParse.pl ###########
### A Perl script that converts a fasta file for use in UCHIME
### For UCHIME step in metabarcoding analysis

### Call: UchimePreParse.pl <filename>
### Input: fasta file with query sequences, aligned

# Input filename
$file = $ARGV[0];

# Path is split into pieces to allow later renaming and saving of copies
if ($file !~  m/\//) {$file = ".\/" . $file;} # si no se da el path
$position = rindex($file, "\/");
$path = substr($file, 0, $position+1);
$name = substr($file, $position+1);
$extposition = rindex($name, "\.");
$ext = substr($name, $extposition+1);
$namenoext = substr($name, 0, $extposition);
print ("\nFilename: $name\n");

# Open the input file
open(FILE, $file)  or die "Can't open file";

# Create an output file and open it; create temporary files for later modification
$newfile = $file . "u1.fas";
open (FILE2, "> $newfile");

# Modify the format to fit with UCHIME
while (<FILE>) {
		chomp;
		if ($_ =~ />.+$/g) {
				$_ =~ s/_/;size=/g;
				$_ =~ s/;size=seq/_seq/g;
				$_ =~ s/\n/;\n/g;
				print FILE2 ($_, ";\n");
				}
		else {
				print FILE2 ($_, "\n");
				}
		}


# Close files
close(FILE);
close(FILE2);
```

```
#!/usr/bin/perl
#use warnings;
```

```
######### UchimePostParse.pl ###########
### A Perl script that returns fasta files to original format after UCHIME run
### For UCHIME step in metabarcoding analysis

### Call: UchimePostParse.pl <filename>
### Input: fasta file with query sequences, aligned

# Input filename
$file = $ARGV[0];

# Path is split into pieces to allow later renaming and saving of copies
if ($file !~  m/\//) {$file = ".\/" . $file;} # si no se da el path
$position = rindex($file, "\/");
$path = substr($file, 0, $position+1);
$name = substr($file, $position+1);
$extposition = rindex($name, "\.");
$ext = substr($name, $extposition+1);
$namenoext = substr($name, 0, $extposition);
print ("\nFilename: $name\n");

# Open the input file
open(FILE, $file)  or die "Can't open file";

# Create an output file and open it; create temporary files for later modification
$newfile = $file . "u2.fas";
open (FILE2, "> $newfile");

# Modify the format to fit with steps after UCHIME
while (<FILE>) {
		chomp;
		$_ =~ s/\n//g;
		$_ =~ s/;size=/_/g;
		$_ =~ s/>/\n>/g;
		$_ =~ s/;/\n/g;
		print FILE2 ($_);
		}

# Close files
close(FILE);
close(FILE2);
```

```
#!/usr/bin/perl
use warnings;

######### DeMultiUchim.pl ###########
### A Perl script that de-multiplexes Uchimera fasta output files

### Call: DeMultiUchim.pl <concatenated fasta file> <sample identifier>
### Input fasta resulting from concatenated analysis of all samples in Uchimera
### Output: Fasta file containing only sequences of desired sample (de-multiplexed)

# Input concatenated fasta file
$file = $ARGV[0];

# Input sample name
$sample = $ARGV[1];

# Path is split into pieces to allow later renaming and saving of copies
if ($file !~  m/\//) {$file = ".\/" . $file;} # if no path is given
$position = rindex($file, "\/");
$path = substr($file, 0, $position+1);
$name = substr($file, $position+1);
$extposition = rindex($name, "\.");
$ext = substr($name, $extposition+1);
$namenoext = substr($name, 0, $extposition);
print ("\nFilename: $name\n");

# Open the input file
open(FILE, $file)  or die "Can't open file";

# Create an output file and open it; create a temporary file for later modification
$newfile = $path . $sample . "u.fas";
$temp1 = $sample . "1.tmp";
$temp2 = $sample . "2.tmp";
open (FILE2, "> $temp1");

# Sum fasta lines
while (<FILE>) {
		if ($_ =~ /^>/g) {
				s/\n/;/g;
				}
		print FILE2 ($_);
		}

# Change temp files from input to output etc.
close(FILE);
close(FILE2);
open (FILE2, "< $temp1");
open (FILE3, "> $temp2");

# Extract all sequences that match the specific sample
while (<FILE2>) {
```

```
        if ($_ =~ />$sample/g) {
                print FILE3 ($_);
                }
        }

# Change temp files from input to output etc.
close(FILE2);
close(FILE3);
open (FILE3, "< $temp2");
open (FILE4, "> $newfile");

# Return sequences to fasta format
while (<FILE3>) {
        $_ =~ s/;/\n/g;
        print FILE4 ($_);
        }

# Close files
close(FILE2);
close(FILE3);
close(FILE4);

# Delete temp files
unlink $temp1;
unlink $temp2;
```

```
#!/usr/bin/perl
#use warnings;


######### parse_jplace.pl ###########

### A Perl script that parses jplace output files to produce result tables
### For joint metabarcoding analysis

foreach $file (@ARGV) {

	if ($file !~  m/\//) {$file = ".\/" . $file;} # si no se da el path
	$position = rindex($file, "\/");
	$path = substr($file, 0, $position+1);
	$name = substr($file, $position+1);
	$extposition = rindex($name, "\.");
	$ext = substr($name, $extposition+1);
	$namenoext = substr($name, 0, $extposition);
	print ("Filename: $name\n");

	open(FILE, $file)  or die "Can't open file";

	$newfile = $path . "/" . $namenoext . "_2." . $ext;

	open (FILE2, "> $newfile");

	$y = 0;
	$order="";
	while (<FILE>) {
		chomp;
		$y++;
		if ($y == 1) {print FILE2 ("Sample\tCluster\tReads\t$_\tOther species\tOrder\n");next;}

		@words = split;

		if ($words[1] == 1) {

			if ($y > 2) {
				print FILE2 ("\t$order\n");
				$order="";
			}
			@words2 = split (/_/, $words[0]);

			print FILE2 ("$words2[0]\t$words2[1]\t$words2[2]\t$_\t");
		}
		else {
			print FILE2 ("$words[10],");
		}

		@words3 = split (/_/, $words[10]);
		$num = map (/$words3[0]/g, $order);
```

```
                if ($num == 0) {
                        $order = $order . $words3[0] . ",";
                }

        }
        print FILE2 ("\t$order\n");
        close(FILE2);

        close(FILE);

}
```

```
#!/usr/bin/perl
#use warnings;


######### fasta2phy.pl  ###########
### A Perl script to convert fasta files into phylip interleaved format with 50 charaters in name

foreach $file (@ARGV) {

	if ($file !~  m/\//) {$file = ".\/" . $file;}

	$position = rindex($file, "\/");
	$path = substr($file, 0, $position);
	$name = substr($file, $position+1);
	$extposition = rindex($name, "\.");
	$ext = substr($name, $extposition+1);
	$namenoext = substr($name, 0, $extposition);
	print ("\nFilename: $name  ");

	open(FILE, $file);
	@array = <FILE>;
	close (FILE);

	$totsp = 0;
	foreach (@array) {
		if(/\>/){
			if ( ( $totsp > 1 ) && ( !($totll == $totll2) ) ) {
				print ("\n\nNot all sequences have the same length \n\n");
			}

			$totll2 = $totll;
			$totll = 0;
			$totsp = $totsp + 1;
			next;
		}
		s/\*//g;
		s/ //g;
		chomp;
		$ll = length();
		$totll = $totll + $ll;

	}

	print("\nNumber of species and sequences: $totsp $totll  \n");

	open(FILE, $file);
	@array = <FILE>;
	close (FILE);
```

```
        $newfile = $path . "/" . $namenoext . ".phy";
        open (FILE2, "> $newfile");
        print FILE2 ("$totsp $totll\n");
        print ("Result in: $newfile\n\n");

        foreach (@array) {
                chomp;
                if (/\>/) {
                        print FILE2  ("\n");
                        s/ /-/g;
                        s/,/-/g;
                        s/;/-/g;
                        s/\./-/g;
                        s/\(/-/g;
                        s/\)/-/g;
                        s/-+/-/g;
                        $firstline = substr($_, 1, 100);
                        printf FILE2 "%-100s ", $firstline;
                }
                else {
                        print FILE2 ($_);
                }

        }
        print FILE2  ("\n");
        close (FILE2);


}
```

D **Plot of genetic divergence and ID score, including only target sequences.**

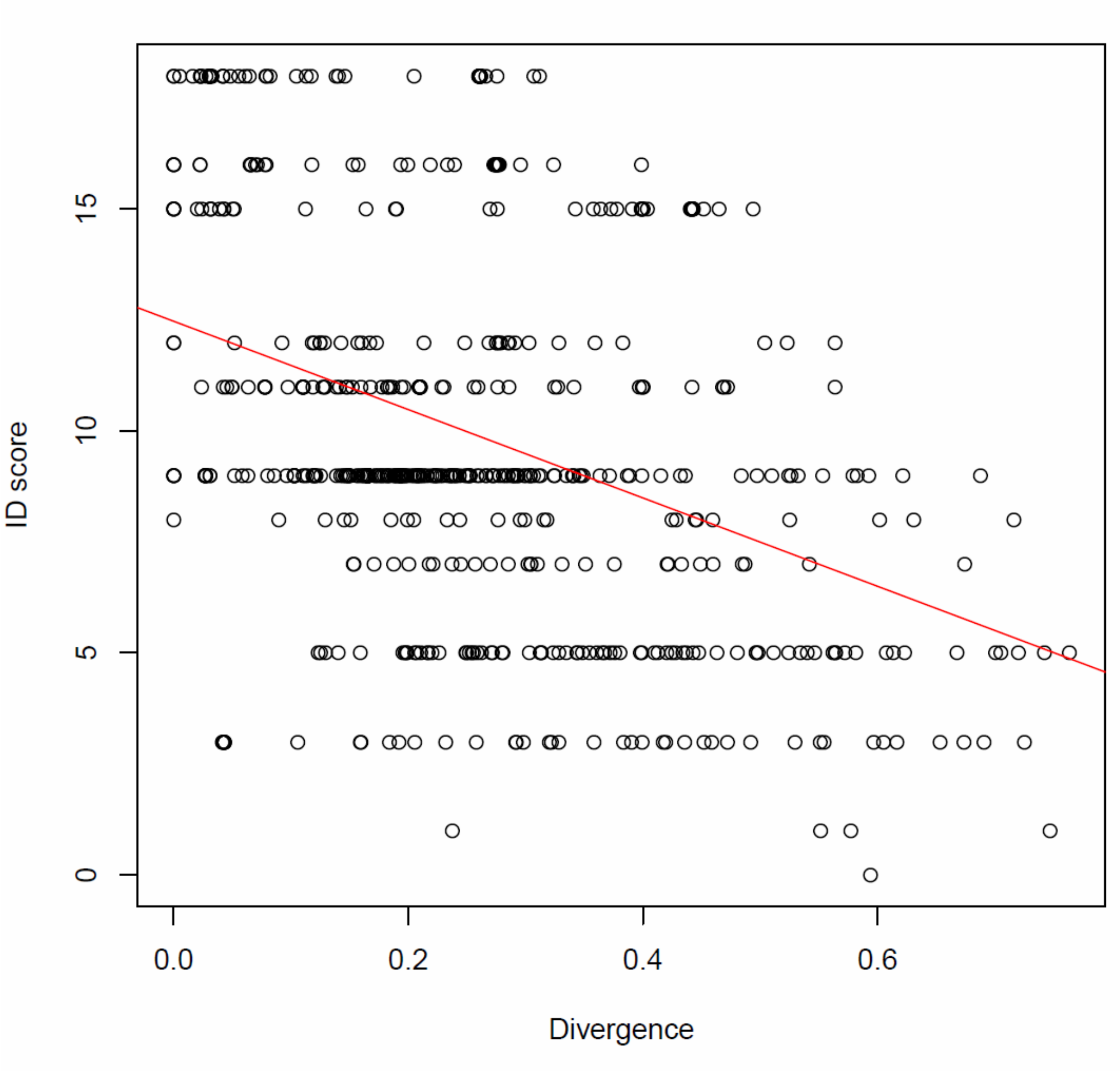

E Summary of the results of BOLD and GenBank blast identification of non-target reads.

Results are given as 1 = present, - = absent, for BOLD / GenBank blast.

	Fresh?	Oomycota: Saprolegni	Oomycota: Phytophtho	Oomycota: Pythium	Oomycota (other)	Ascomycota	Fungi (other)	Protista	Plants (algae)	Rotifera	Hemiptera (terrestrial)	Collembola + Protura	Acari	Other Arthropod	Other
**BC0023**	yes	-/-	1/1	-/-	-/-	-/-	-/-	-/-	-/-	-/-	1/1	-/-	-/-	-/-	-/-
**BC0024**	yes	-/-	1/-	1/1	-/-	-/-	-/-	-/-	-/-	1/1	1/1	-/-	-/-	-/-	-/-
**BC0028**	yes	-/-	1/-	1/1	-/-	-/-	-/-	-/-	-/-	1/1	1/1	1/1	1/1	1/1	1/-
**BC0041**	yes	-/-	-/-	-/-	-/-	-/-	-/-	-/-	-/-	1/1	1/1	-/-	-/-	1/-	-/-
**BC0061**	yes	-/-	1/-	1/1	-/-	-/-	-/-	-/-	-/1	-/1	1/1	-/-	-/-	-/-	-/-
**BC0240**	yes	-/-	-/-	-/-	-/-	-/-	-/-	-/-	-/-	-/-	-/-	-/1	-/-	1/-	-/-
**BC0293**	yes	-/-	-/-	-/-	-/-	1/1	-/-	-/1	-/-	-/-	1/1	-/-	-/-	-/-	-/-
**BC0648**	no	1/1	-/-	1/1	-/-	-/-	-/-	-/-	-/-	-/-	-/-	-/-	-/-	-/-	-/-
**BC0780**	no	-/-	-/-	-/-	-/-	1/1	-/-	-/-	1/1	1/1	-/1	-/-	-/-	-/-	-/1
**BC0834**	no	-/-	-/-	-/-	-/-	1/1	-/-	-/1	1/-	-/1	-/-	-/-	-/-	-/1	1/-
**BC0849**	no	-/-	1/-	-/-	1/-	-/1	-/-	-/1	-/-	1/1	-/1	-/-	-/-	1/-	-/-
**BC0857**	no	-/-	-/-	1/1	-/-	-/-	-/-	1/-	-/-	-/-	1/1	-/-	-/-	-/-	-/-
**BC0861**	no	1/1	1/1	-/-	-/-	-/1	1/1	-/1	1/-	1/1	-/-	-/1	1/-	1/-	1/1
**BC0868**	yes	-/-	-/-	-/-	-/-	-/-	-/-	-/-	-/-	1/1	-/-	-/-	-/-	-/1	1/-
**BC0873**	no	1/1	-/-	-/1	-/-	-/-	-/-	-/-	-/-	1/1	1/1	-/-	-/-	-/-	-/-
**BC0877**	no	1/1	-/-	1/1	-/-	-/-	-/-	-/-	-/1	1/1	-/-	-/-	-/-	-/1	1/-
**BC0899**	no	1/1	1/1	1/1	-/-	-/-	1/-	-/1	-/1	1/1	-/-	-/-	-/-	1/1	1/-
**BC0917**	no	-/-	1/1	-/-	-/-	-/-	-/-	-/-	1/1	1/1	-/-	-/-	-/-	-/1	-/-
**BC0939**	no	-/-	-/-	-/1	-/-	-/-	-/-	-/-	-/1	1/1	1/1	-/-	-/-	-/-	-/-
**BC0943**	no	-/-	1/1	1/-	-/-	-/-	-/-	-/-	-/1	-/-	-/-	-/-	-/-	1/1	1/1
**BC0974**	no	-/-	-/-	-/-	-/-	1/1	-/-	-/-	1/1	-/1	-/-	-/-	-/1	1/1	1/-
**BC0981**	no	-/-	1/-	1/1	-/-	-/-	1/1	-/-	1/1	1/1	-/-	-/-	-/-	-/-	-/-
**BC1035**	yes	-/-	1/1	-/-	1/-	-/-	1/-	-/1	1/1	1/1	-/-	-/1	1/-	1/-	-/1
**BC1041**	yes	-/-	-/-	1/1	-/-	1/1	-/-	-/-	-/-	1/1	-/-	-/-	-/-	-/1	1/-
**BC1059**	yes	-/-	1/1	-/1	1/-	-/-	-/-	-/-	-/-	-/-	-/-	-/-	-/1	1/-	-/-
**BC1062**	yes	-/-	1/1	1/1	1/1	-/-	-/-	-/-	1/1	1/1	-/-	-/-	-/-	-/-	-/-
**BC1101**	yes	-/-	-/-	-/-	-/-	-/-	-/-	-/-	-/1	-/-	-/-	-/1	-/-	-/-	-/1
**BC1108**	yes	-/-	1/-	-/-	-/-	-/-	-/-	-/-	-/-	-/-	-/-	-/-	-/-	1/-	-/1
**BC1123**	yes	-/-	-/-	-/-	-/-	-/-	-/-	-/1	-/-	1/1	1/-	-/-	-/-	1/1	-/-
**BC1144**	yes	-/-	1/1	-/-	-/-	-/-	-/-	-/-	-/-	-/-	-/-	-/-	-/-	-/-	-/-
**BC1150**	yes	-/-	1/1	1/1	1/1	-/1	-/-	-/-	-/-	1/1	-/-	-/-	-/-	-/-	-/-
**BC1154**	yes	-/-	1/1	-/-	1/1	-/1	-/-	-/1	1/1	1/1	-/-	-/-	-/-	-/1	1/1

**BC1243**	yes	-/-	-/-	-/-	-/-	-/-	-/-	-/1	-/1	-/-	-/-	-/-	1/-	-/-	-/-
**C1131**	no	-/-	1/-	-/-	-/-	-/-	-/-	-/-	-/-	-/-	-/-	-/-	-/-	-/-	-/-
**C1654**	no	-/-	-/-	-/-	-/-	1/1	-/-	-/-	-/-	-/-	-/-	-/1	-/-	1/1	1/-
**C1661**	no	-/-	-/-	-/-	-/-	-/-	-/-	-/-	-/-	-/-	-/-	-/-	-/-	1/1	1/-
**C1671**	no	-/-	-/-	-/-	-/-	1/1	-/-	-/-	-/-	-/-	-/-	-/1	-/-	-/-	-/-
**C1796**	no	1/1	-/-	-/-	-/-	-/-	-/-	-/-	-/-	1/1	1/1	-/-	-/-	-/-	-/-
**C3855**	no	-/-	1/1	1/1	1/-	-/-	-/-	-/1	1/1	-/-	-/-	-/1	1/-	1/-	-/-
**C4323**	no	-/-	-/-	-/-	-/-	1/1	-/-	-/1	-/-	-/-	-/-	-/-	-/-	1/-	-/-
**OHGC001**	no	1/1	-/-	1/1	-/-	-/-	-/-	-/1	-/-	-/-	-/-	-/-	1/1	1/-	-/-
